# Supplementary material for: scPrediXcan integrates deep learning methods and single-cell data into a cell-type-specific transcriptome-wide association study framework
Source: Cell Genom. 2025 May 14;5(5):100875. doi: 10.1016/j.xgen.2025.100875 (PMC12143334; doi:10.1016/j.xgen.2025.100875)
Supplement: Document S1. Figures S1–S10 [file mmc1.pdf]

Cell Genomics, Volume *5*

Supplemental information

scPrediXcan integrates deep learning methods and single-cell data into a cell-type-specific transcriptome-wide association study framework

Yichao Zhou, Temidayo Adeluwa, Lisha Zhu, Sofia Salazar-Magaña, Sarah Sumner, Hyunki Kim, Saideep Gona, Festus Nyasimi, Rohit Kulkarni, Joseph E. Powell, Ravi Madduri, Boxiang Liu, Mengjie Chen, and Hae Kyung Im

Supplemental information

scPrediXcan integrates deep learning methods and single-cell data into a cell-type–specific transcriptome-wide association study framework

Yichao Zhou, Temidayo Adeluwa, Lisha Zhu, Sofia Salazar-Magaña, Sarah Sumner, Hyunki Kim, Saideep Gona, Festus Nyasimi, Rohit Kulkarni, Joseph Powell, Ravi Madduri, Boxiang Liu, Mengjie Chen, Hae Kyung Im

Figure S1: ctPred predicts cell-type-specific gene expressions in CD 4+ T cell

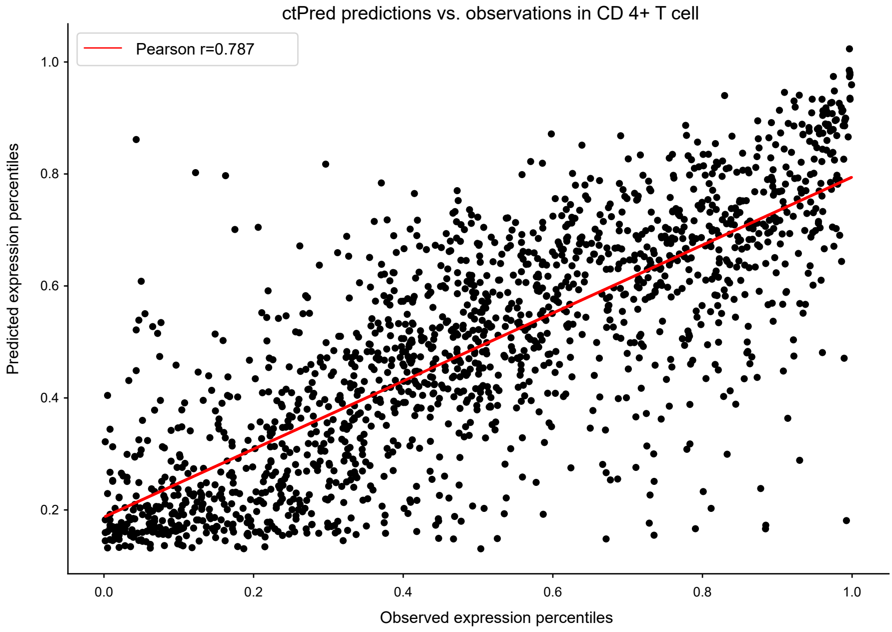

Figure S1 This supplemental figure is related to main figure 2. Scatter plot of ctPred predictions and observations for gene expression percentiles in CD4+ T cell dataset.

Figure S2: Quantile-quantile plot of ACAT-adjusted TWAS -$\log_{10}$ (p-value) against uniformly distributed p-value for T2D and SLE.

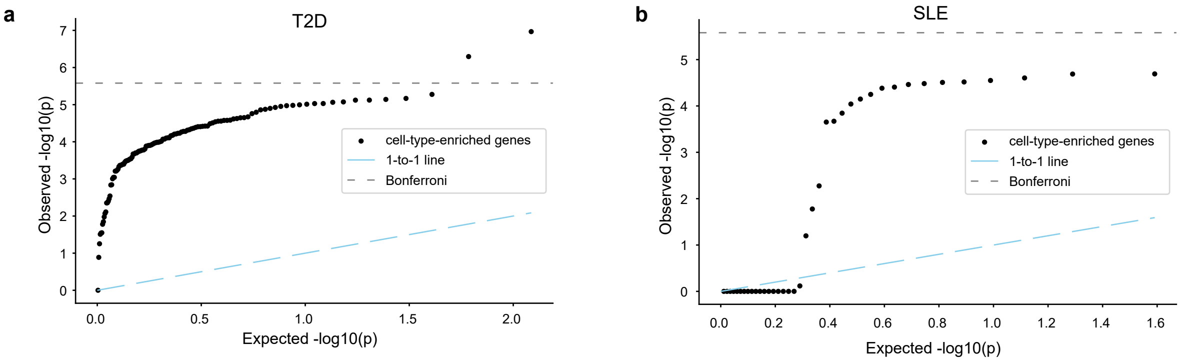

Figure S2 This supplemental figure is related to main figure 5e and 6c. **a)** Quantile-quantile plot of ACAT-aggregated TWAS -log10 (p-value) in all non-significant cell types for genes passing the Bonferroni-corrected threshold in only one islet cell type from T2D dataset for T2D trait. **b)** Quantile-quantile plot of ACAT-aggregated TWAS -log10 (p-value) in all non-significant cell types for genes passing the Bonferroni-corrected threshold in only one immune cell type from OneK1K dataset for SLE trait.

Figure S3: Calculating the number of true positive genes with p-values deviating from a uniform distribution.

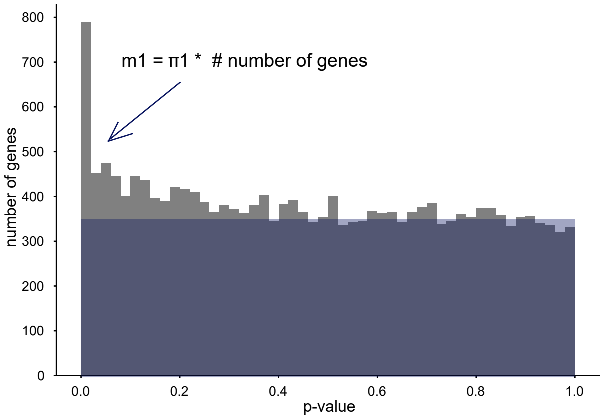

Figure S3 This supplemental figure is related to main figure 3d, e. The m1 calculation is based on the histogram of p-values of Pearson correlations between predicted gene expressions and observed gene expressions. The blue rectangular region shows the genes with p-values following a uniform distribution, and the proportion
of those genes is denoted as 0. The 1 is 1-0, and the number of true positive genes is calculated by 1 * total number of genes.

Figure S4: Distribution of Spearman correlations between ctPred predictions and l-ctPred predictions of all genes in representing cell types

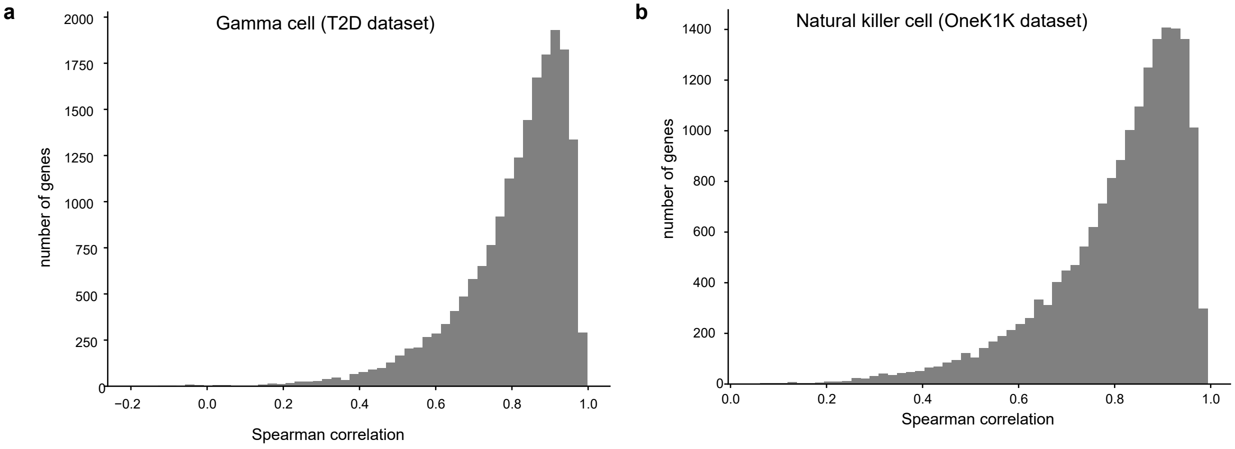

Figure S4 This supplemental figure is related to main figure 4. **a)** Histogram of Spearman correlations between ctPred- and l-ctPred-predicted gene expression percentiles in gamma cell as the representing cell type from T2D dataset. Other cell types have similar correlation distributions. **b)** Histogram of Spearman correlations between ctPred-predicted gene expressions and ℓ-ctPred-predicted gene expressions in natural killer cells as the representing cell type from OneK1K dataset. Other cell types have similar correlation distributions.

Figure S5: Quantile-quantile plot of TWAS -$\log_{10}$ (p-value) for only the overlapped genes between models

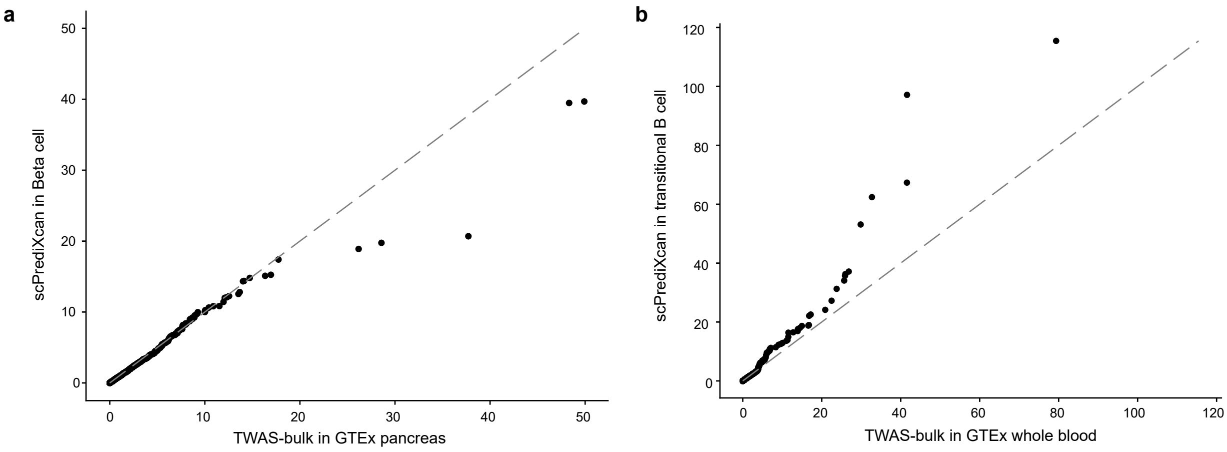

Figure S5 This supplemental figure is related to main figure 5c, 6b. **a)** Quantile-quantile plot of T2D TWAS -log10(p) of overlapped genes in scPrediXcan in Beta cell and TWAS-bulk in GTEx pancreas. This set of genes will likely favor the TWAS-bulk method since only models that performed well enough in this approach end up included here. A fairer comparison is shown in Figure 5b where union of genes tested by scPrediXCan and TWAS-bulk in GTEx pancreas are shown, imputing the p-values of genes missed by TWAS-bulk with uniformly distributed p-values. **b)** Quantile-quantile plot of SLE TWAS -log10(p) of overlapped genes in scPrediXcan in transitional B cell and TWAS-bulk in GTEx blood.

Figure S6: ctPred prediction performance metrics vs. cell numbers and read counts of different cell types across datasets.

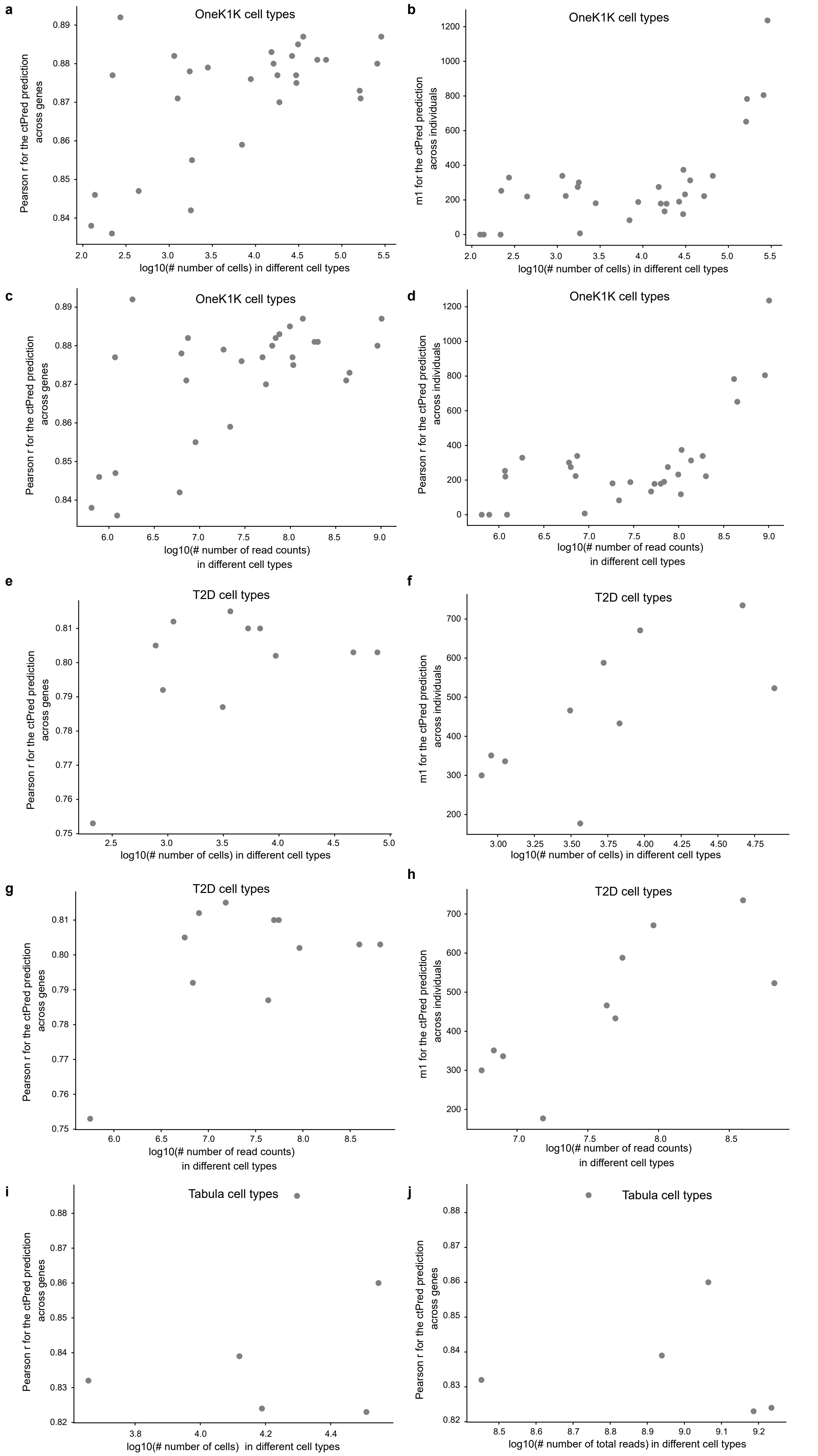

Figure S6 This supplemental figure is related to main figure 3c-e. **a)-d)** Prediction performance vs number of cells and read counts in OneK1K dataset. Scatter plot of ctPred prediction metrics (Pearson r for prediction across genes or m1 value for prediction across individuals) and cell numbers or total scRNA-seq read counts in different cell types from OneK1K dataset. **e)-h)** Prediction performance vs number of cells and read counts in T2D dataset. Scatter plot of ctPred prediction metrics (Pearson r for prediction across genes or m1 value for prediction across individuals) and cell numbers or total scRNA-seq read counts in different cell types from T2D dataset. **i)-j)** Prediction performance vs number of cells and read counts in Tabula Sapiens subset. Scatter plot of ctPred prediction metrics and cell numbers or total scRNA-seq read counts in different cell types from Tabula Sapiens subset.

Figure S7: scRNA-seq pseudobulk data processing for ctPred training.

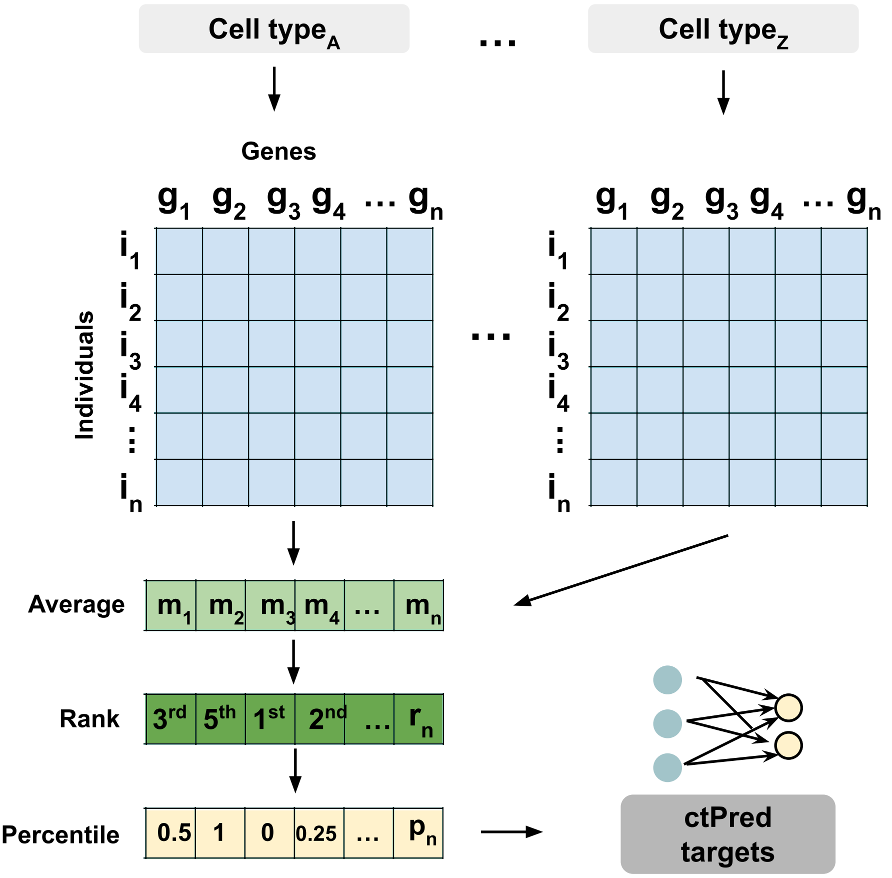

Figure S7 This supplemental figure is related to main figure 1. The scRNA-seq processing into the target for ctPred model training.

Figure S8: Comparison between l-ctPred and PEN in predicting gene expression across individuals in different cell types.

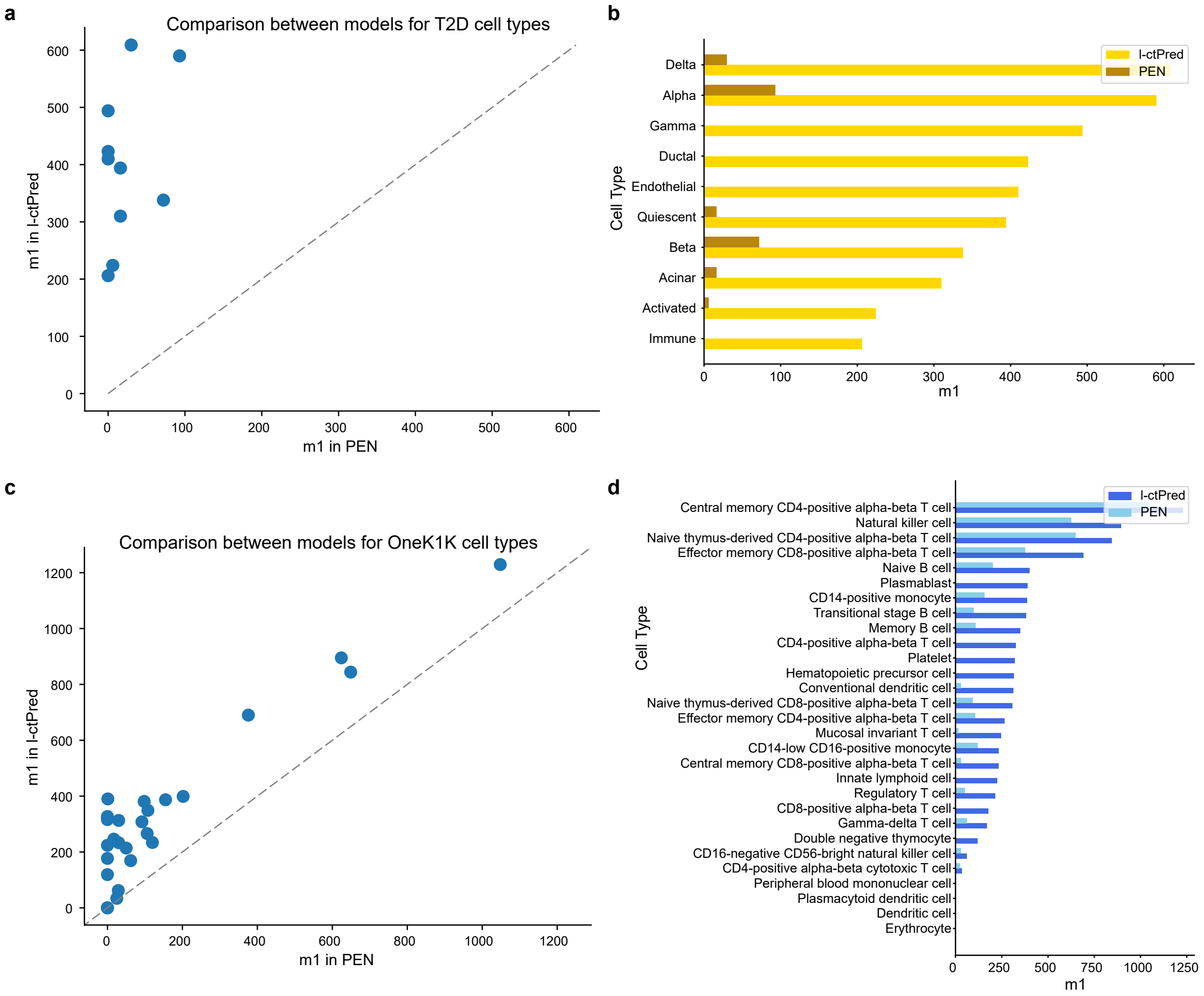

Figure S8 This supplemental figure is related to main figure 3d-e. **a)** Scatter plot of m1 values for ℓ-ctPred and PEN in predicting gene expression percentiles across individuals for T2D cell types. Each dot is a cell type. **b)** Bar plot of m1 values for ℓ-ctPred and PEN in predicting gene expression percentiles across individuals for T2D cell types. **c)** Scatter plot of m1 values for ℓ-ctPred and PEN in predicting gene expression percentiles across individuals for OneK1K cell types. Each dot is a cell type. **d)** Bar plot of m1 values for ℓ-ctPred and PEN in predicting gene expression percentiles across individuals for OneK1K cell types.

Figure S9: Shapley value feature importance analysis of ctPred in T-cell.

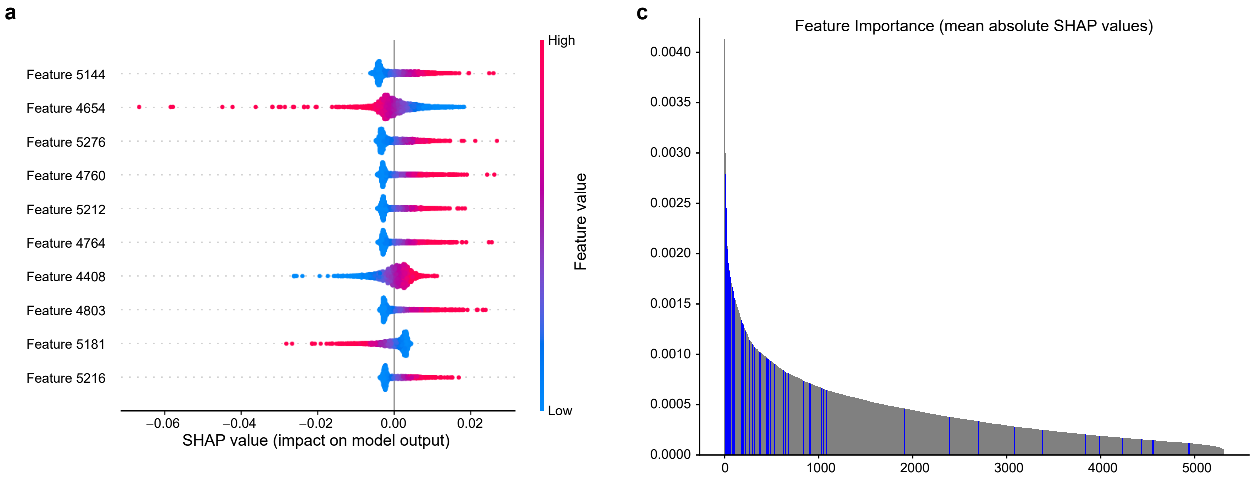

b

Rank	Feature index	Feature description
1	5144	CAGE:Mast cell
2	4654	CHIP:ATF4:ChIP-seq, InVitroCistromics_ATF4_0.02uL_plusCEBPb_0.1uL / hMSC / Human Mesenchymal Stem Cells
3	5276	CAGE:Mast cell, expanded
4	4760	CAGE:CD8+ T Cells
5	5212	CAGE:CD4+CD25+CD45RA- memory regulatory T cells expanded
6	4764	CAGE:Natural Killer Cells
7	4408	CHIP:H3K36me3:vagina female adult (53 years),reproductive system,none,vagina,mesoderm
8	4803	CAGE:Mast cell - stimulated
9	5181	CAGE:testis, adult, pool2
10	5216	CAGE:CD4+CD25-CD45RA- memory conventional T cells expanded
11	4383	CHIP:H3K9me3:ovary female adult (30 years),reproductive system
12	4091	CHIP:H3K36me3:thyroid gland female adult (53 years),endocrine system
13	5211	CAGE:CD4+CD25+CD45RA+ naive regulatory T cells expanded
14	5277	CAGE:Mast cell, expanded and stimulated
15	4521	CHIP:.:batch1_chrom1_LoVo_CASP8AP2_Rabbit_PassedQC / LoVo / colon adenocarcinoma
16	4694	CAGE:testis, adult, pool1
17	4564	CHIP:.:batch1_chrom1_LoVo_NPAT_Rabbit_PassedQC / LoVo / colon adenocarcinoma
18	2137	CHIP:H3K27me3:naive thymus-derived CD4-positive, alpha-beta T cell
19	2576	CHIP:H3K27me3:effector memory CD4-positive, alpha-beta T cell
20	3275	CHIP:eGFP-ZNF512:K562 genetically modified using stable transfection,immune system

Figure S9 This supplemental figure is related to main figure 2b-c. **a)** SHAP summary plot of top10 features for ctPred in predicting gene expression percentiles in T-cell trained from Tabula Sapiens dataset. **b)** Descriptions of top20 impactful features for ctPred in predicting gene expression percentiles in T-cell trained from Tabula Sapiens dataset. **c)** Bar plot of mean absolute Shapley values for all the input features of ctPred in predicting gene expression percentiles in T-cell trained from Tabula Sapiens dataset. Blue: epigenomics features from T-cell. Grey: other epigenomics features.

Figure S10: scPrediXcan in T2D outperforms TWAS method UTMOST.

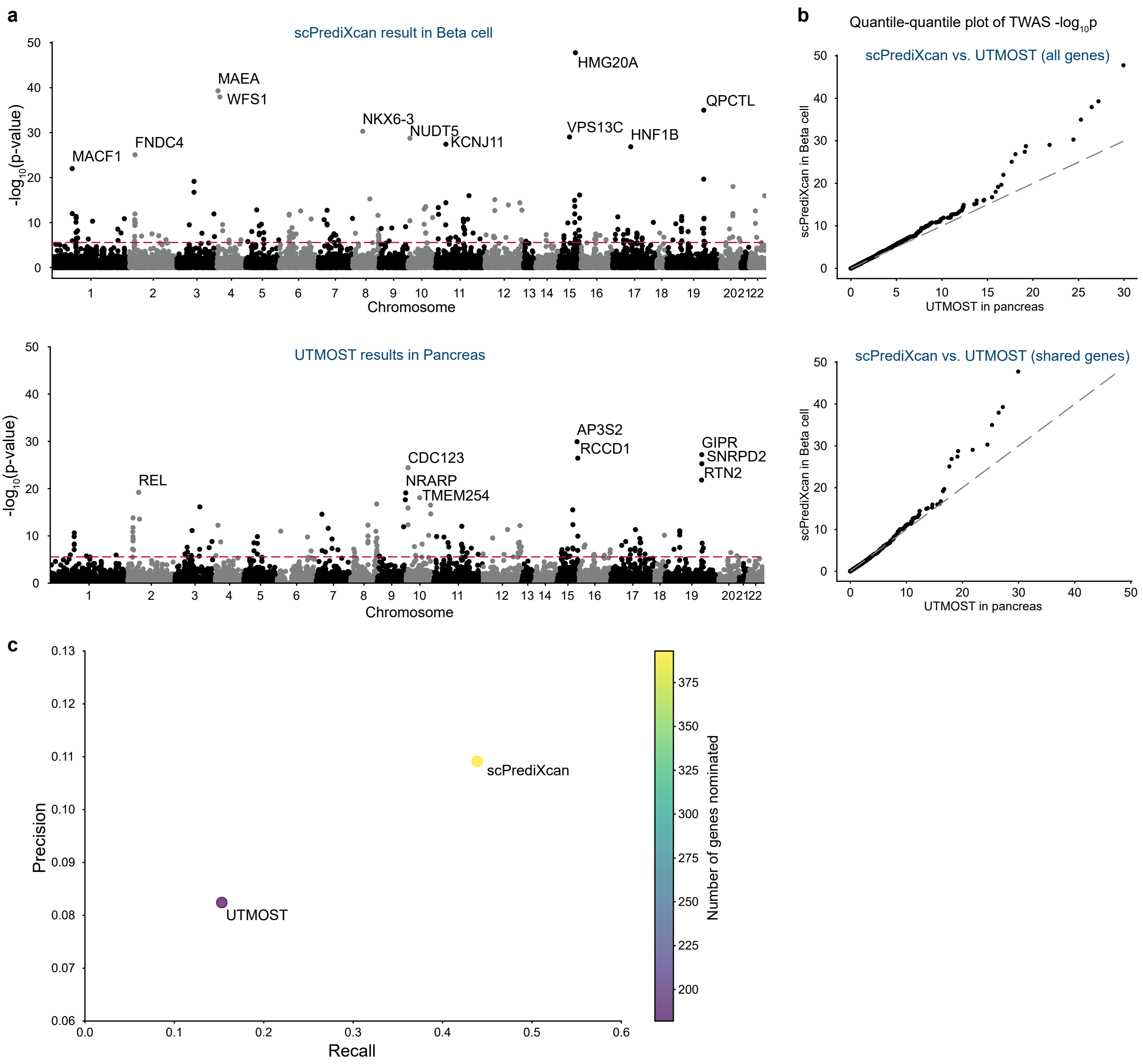

Figure S10 This supplemental figure is related to main figure 5a. **a)** Manhattan plots of T2D TWAS results for different frameworks. Top: scPrediXcan in beta cell from T2D dataset. Bottom: UTMOST in pancreas tissue from GTEx dataset. The red dashed lines are Bonferroni-corrected thresholds ($p<0.05/$ number of genes in the association study). **b)** QQ-plot of TWAS p-values in T2D between frameworks. Shared genes: overlapped genes in both frameworks. All genes: all the protein-coding genes. Considering that ℓ-ctPred achieves convergence for significantly more genes than the SNP-based models used in the other two frameworks, we used a uniform distribution of p-values to represent genes absent in the canonical approaches, ensuring a comprehensive comparison. **c)** Scatter plot of precision and recall between scPrediXcan and UTMOST for T2D causal gene nomination.
